# Supplementary material for: A spliced latency-associated VZV transcript maps antisense to the viral transactivator gene 61
Source: Nat Commun. 2018 Mar 21;9:1167. doi: 10.1038/s41467-018-03569-2 (PMC5862956; doi:10.1038/s41467-018-03569-2)
Supplement: Supplementary file 1 — Supplementary Information(PDF 1685 kb) [file 41467_2018_3569_MOESM1_ESM.pdf]

# Supplementary Information

# A spliced latency-associated VZV transcript maps antisense to the viral transactivator gene 61

Depledge et al.

## Supplementary Figures

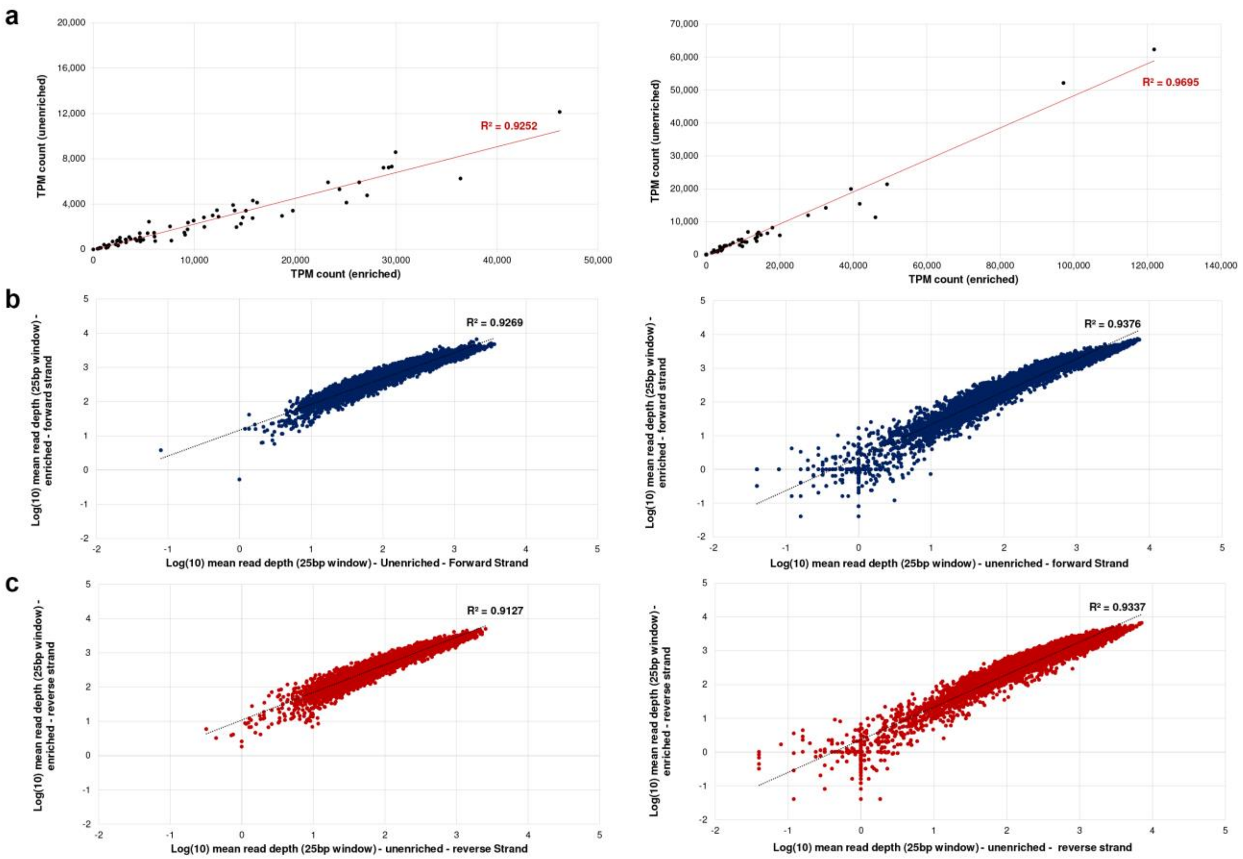


**Supplementary Figure 1 | Unbiased enrichment of VZV and HSV-1 transcriptomes in lytically virus-infected human ARPE-19 cells. a**, Transcript per million (TPM) counts were generated for all canonical ORFs of VZV (left) and HSV-1 (right) in VZV/HSV-1–enriched and unenriched sequence datasets derived from poly-A-selected RNA extracted from lytically virus-infected ARPE-19 cells. $R^2$ correlation scores are shown in red. **b** and **c**, Mean read depths across iterative 25-nt windows for VZV (left) and HSV-1 (right) genomes were plotted using a log scale for VZV/HSV-1–enriched and unenriched sequence datasets derived from poly-A-selected RNA. Forward strand (**b**) and reverse strand (**c**) show a high correlation ($R^2$ > 0.91) between enriched and unenriched datasets.

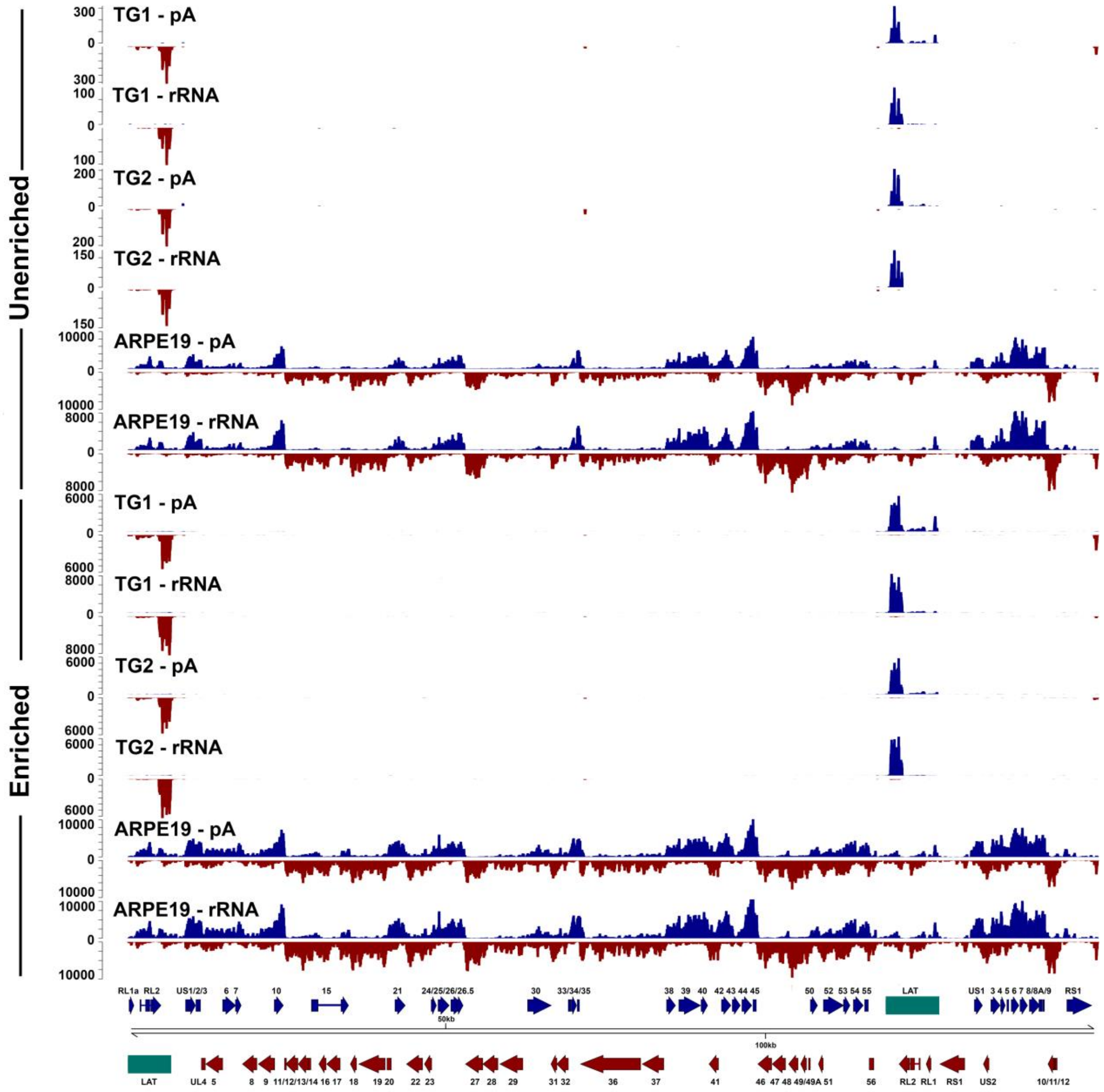


**Supplementary Figure 2 | Comparison of unenriched and enriched poly-A-selected and rRNA-degraded HSV-1 transcriptome in latently HSV-1-infected human TG**. RNA-Seq read data presented in a linear format along the entire 152-kb HSV-1 genome for TG specimens from donors 1 and 2 (Supplementary Table 1) and for lytically HSV-1-infected ARPE-19 cells. Each track depicts either unenriched or enriched poly-A-selected (pA) or rRNA-degraded (rRNA) HSV-1 transcriptome. Blue and red tracks represent read data originating from the sense and antisense strand, respectively. Y-axis values indicate read depth. Previously described HSV-1

ORFs within this locus (blue and red arrows) are shown. Paired-end read datasets were generated with read lengths of 2x34 bp (ARPE-19 cells) or 2x76 bp (TG1 and TG2).

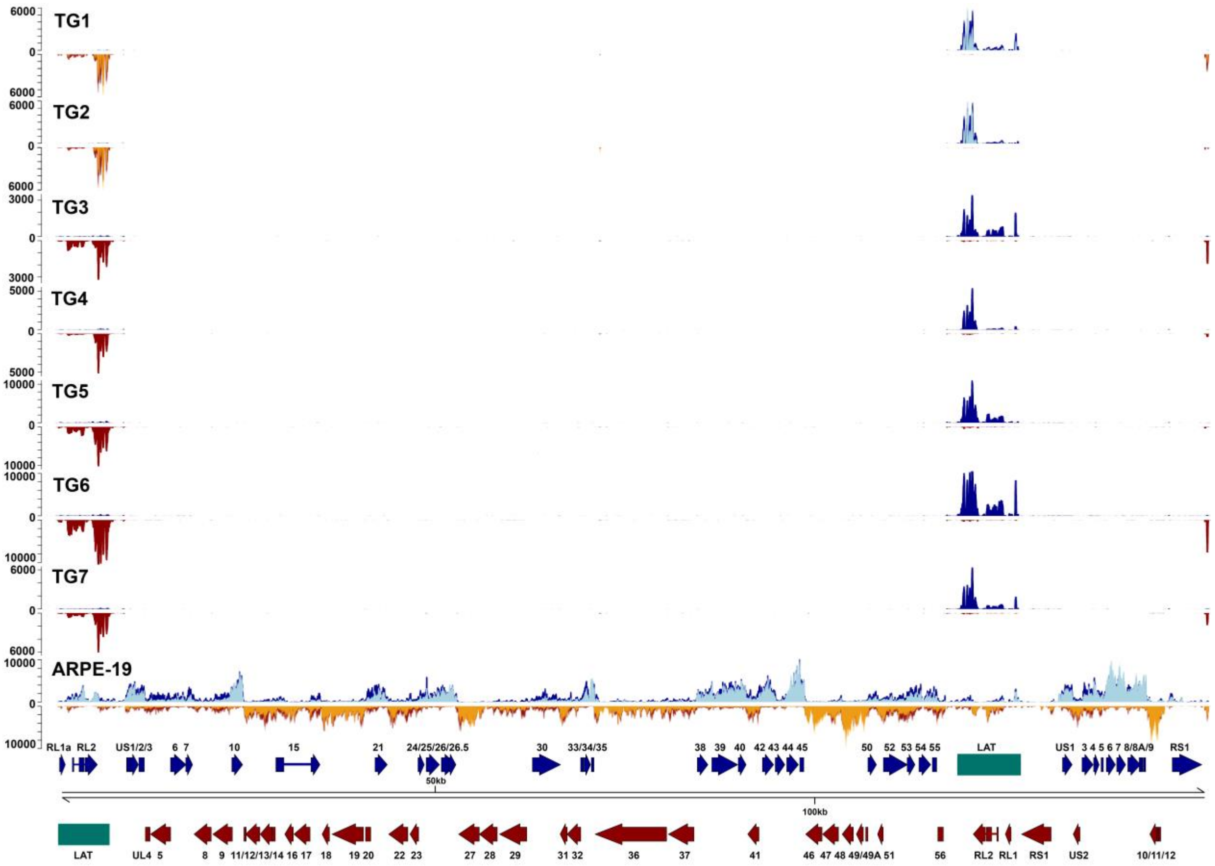


**Supplementary Figure 3 | The latent and lytic transcriptome of HSV-1**. RNA-Seq read data presented in a linear format along the entire 152-kb HSV-1 genome for all seven TG specimens separately and lytically HSV-1-infected ARPE-19 cells. Each track depicts the poly-A-selected, HSV-1 enriched transcriptome. Blue and red tracks represent read data originating from the sense and antisense strand, respectively. Y-axis values indicate read depth. Unenriched tracks superimposed on TG1, TG2 and ARPE-19 are shown in light blue (sense) or orange (antisense). Previously described HSV-1 ORFs are indicated by blue (sense strand) and red (antisense strand) arrows, while the HSV-1 latency-associated transcripts (LATs) are indicated by green boxes. Paired-end read datasets were generated with read lengths of 2x34 bp (ARPE-19), 2x76 bp (TG1 and TG2) or 2x151 bp (TG3 - TG7).

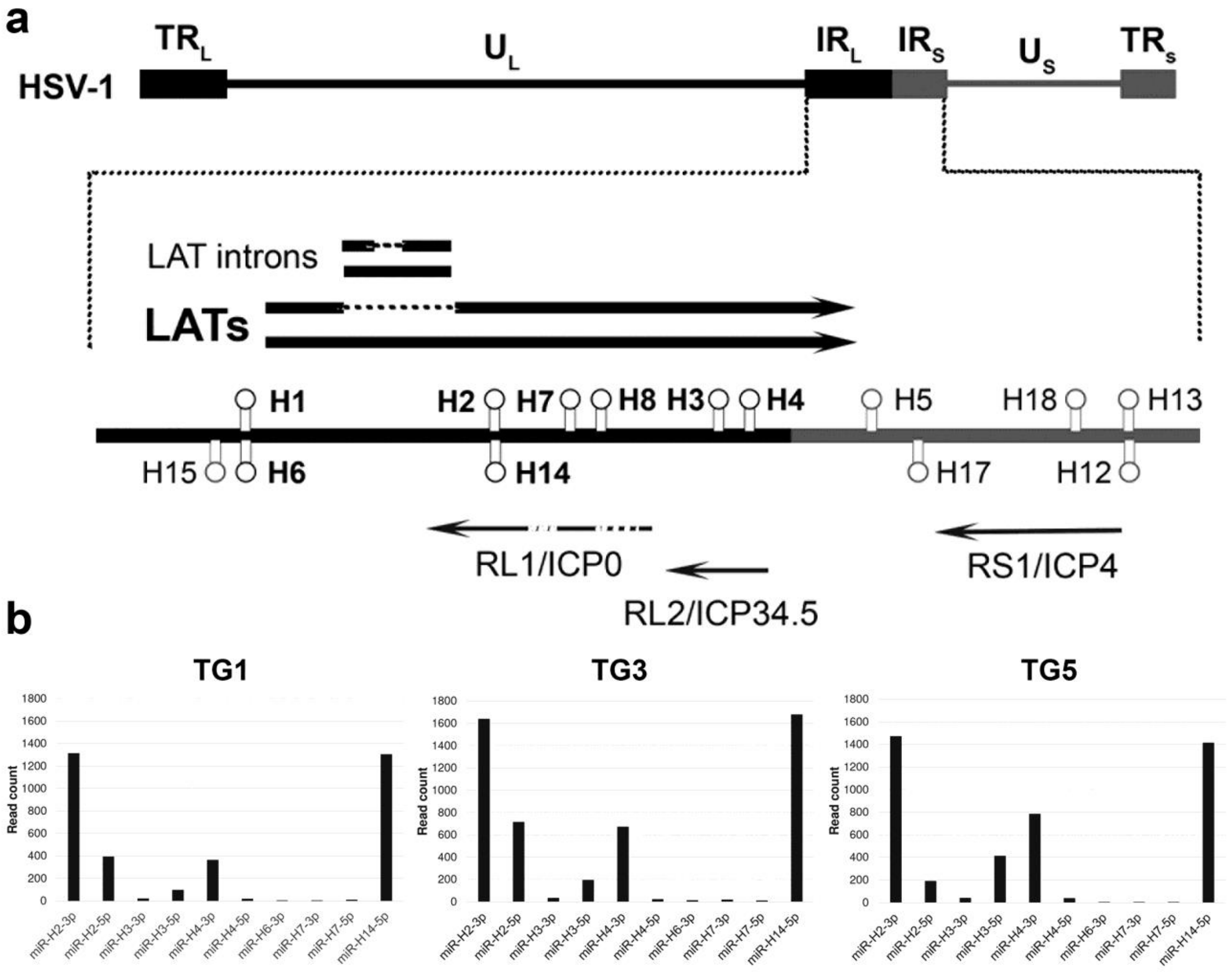


**Supplementary Figure 4 | HSV-1 miRNAs detected in latently HSV-1-infected human TG. a,** Five canonical miRNAs (miR-H2, 3, 4, 7 and 8) are encoded within HSV-1 LAT, while a sixth (miR-H14) is encoded antisense to miR-H7. Two additional miRNAs (miR-H1 and H6) expressed during latency are encoded just upstream of the 5’ end of LAT. **b**, miRNA-Seq of TG1, TG3 and TG5 confirmed transcription of these and the absence of remaining miRNAs encoded in the wider HSV-1 LAT locus (and wider genome, data not shown). Only miRNAs producing >5 reads are shown.

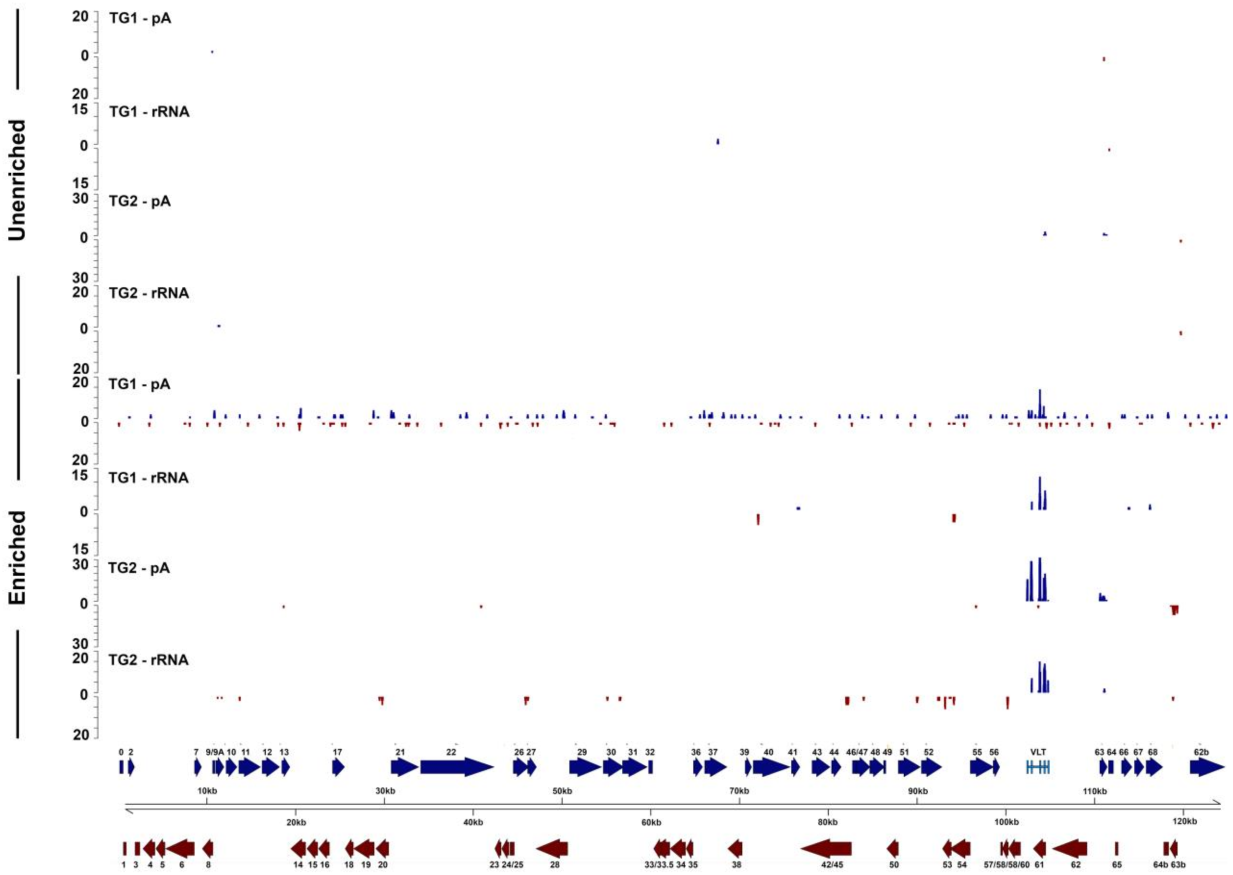


**Supplementary Figure 5 | Comparison of unenriched and enriched poly-A-selected and rRNA-degraded VZV transcriptome in latently VZV-infected human TG**. RNA-Seq read data presented in a linear format along the entire 125-kb VZV genome for TG specimens from donors 1 and 2 (Supplementary Table 1). Each track depicts either an unenriched or enriched poly-A-selected (pA) or rRNA-degraded (rRNA) VZV transcriptome. Blue and red tracks represent read data originating from the sense and antisense strand, respectively. Y-axis values indicate read depth]. Previously described VZV ORFs (blue and red arrows) and the five VLT exons (light blue boxes) were scaled representatively. Paired-end read datasets were generated with read lengths of 2x76 bp.

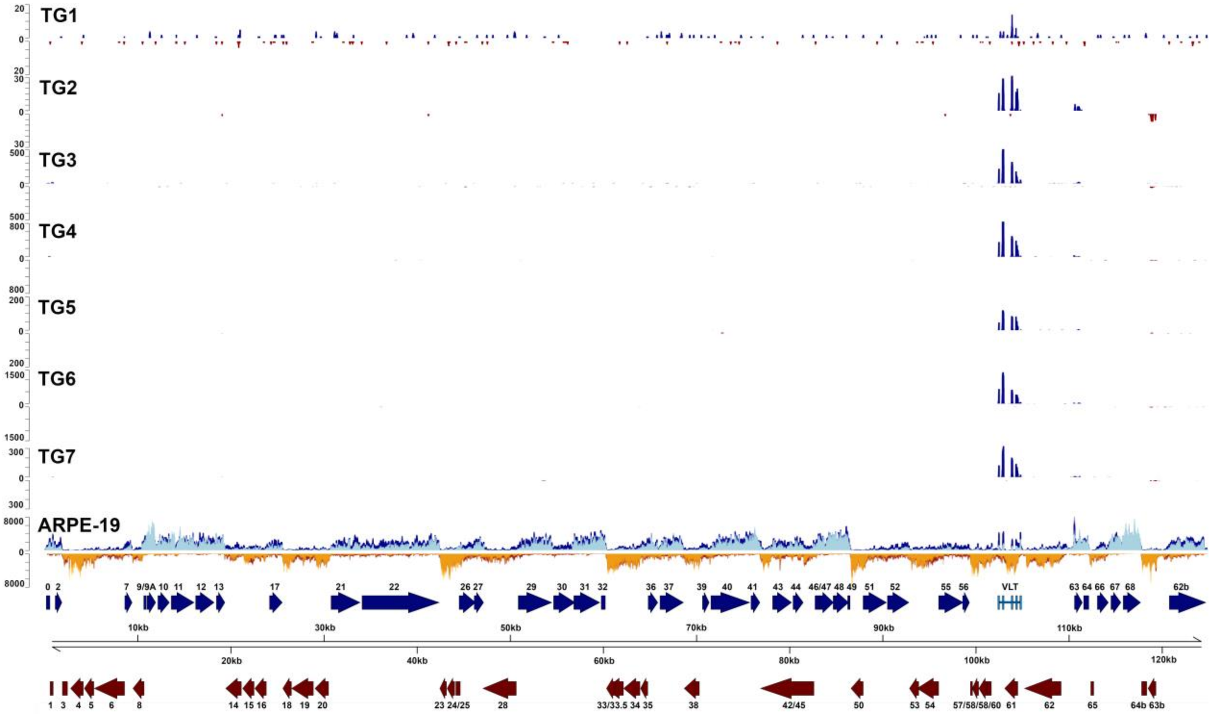


**Supplementary Figure 6 | The latent and lytic transcriptome of VZV**. RNA-Seq read data for seven TG specimens and lytically VZV-infected ARPE-19 cells (Fig. 2), presented in a linear format along the entire 125-kb VZV genome. Each track depicts poly-A-selected, VZV-enriched transcriptomes. Blue and red tracks represent read data originating from the sense and antisense strand, respectively. Y-axis values indicate read depth. Unenriched tracks superimposed on ARPE-19 cells are shown in light blue (sense) or orange (antisense). Note that no VZV-mapping reads were obtained from unenriched sequence datasets generated for TG1 and TG2. Previously described VZV ORFs within this locus (blue and red arrows) and the five VLT exons (light blue boxes) are scaled representatively. Paired-end read datasets were generated with read lengths of 2 x 34 bp (ARPE-19) or 2 x 76bp (TG1 & TG2) or 2 x151bp (TG3 - TG7). VLT was consistently detected in all TGs. Background noise observed in TG1 likely reflects a combination of low sequencing depth and low VZV genome abundance (Supplementary Table 1).

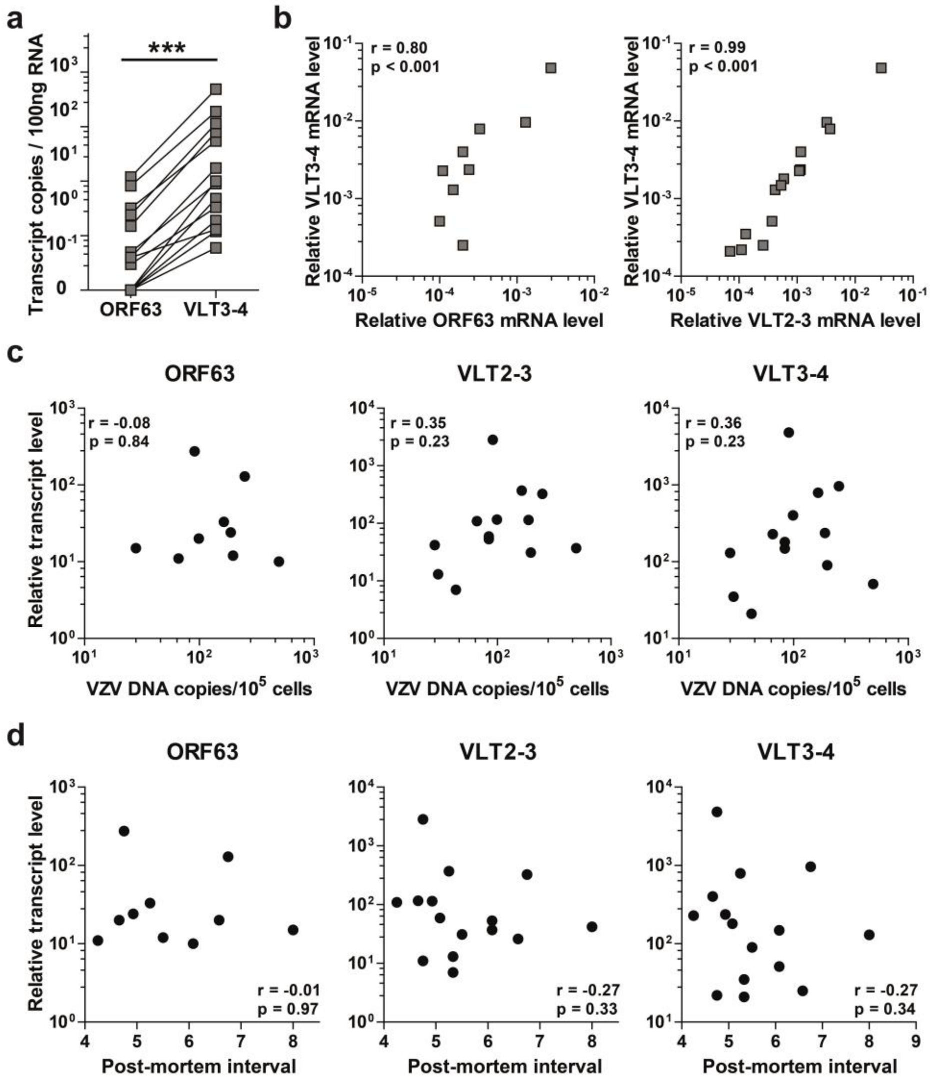


**Supplementary Figure 7 | Prevalence of VZV ORF63 transcript and VLT in human TG. a,** Relative abundance of ORF63 transcript and VLT levels in 15 VZV nucleic acid positive (VZV$^{POS}$) human TG determined by RT-qPCR. VLT2-3 and VLT3-4 refer to primers/probes spanning the VLT exons 2→3 and 3→4 junctions (Supplementary Table 5). ORF63 transcript and VLT levels were paired in the same TG specimens. *** p< 0.001; Wilcoxon signed rank test. **b,** Correlation between relative ORF63 transcript and VLT levels, both normalized to cellular β-actin transcript levels. Spearman correlations are indicated. **c** and **d,** Correlation between relative ORF63 transcript and VLT levels and VZV DNA load (**c**) and post-mortem interval (**d**) by qPCR in 15 VZV$^{POS}$ human TG. Spearman correlations are indicated.

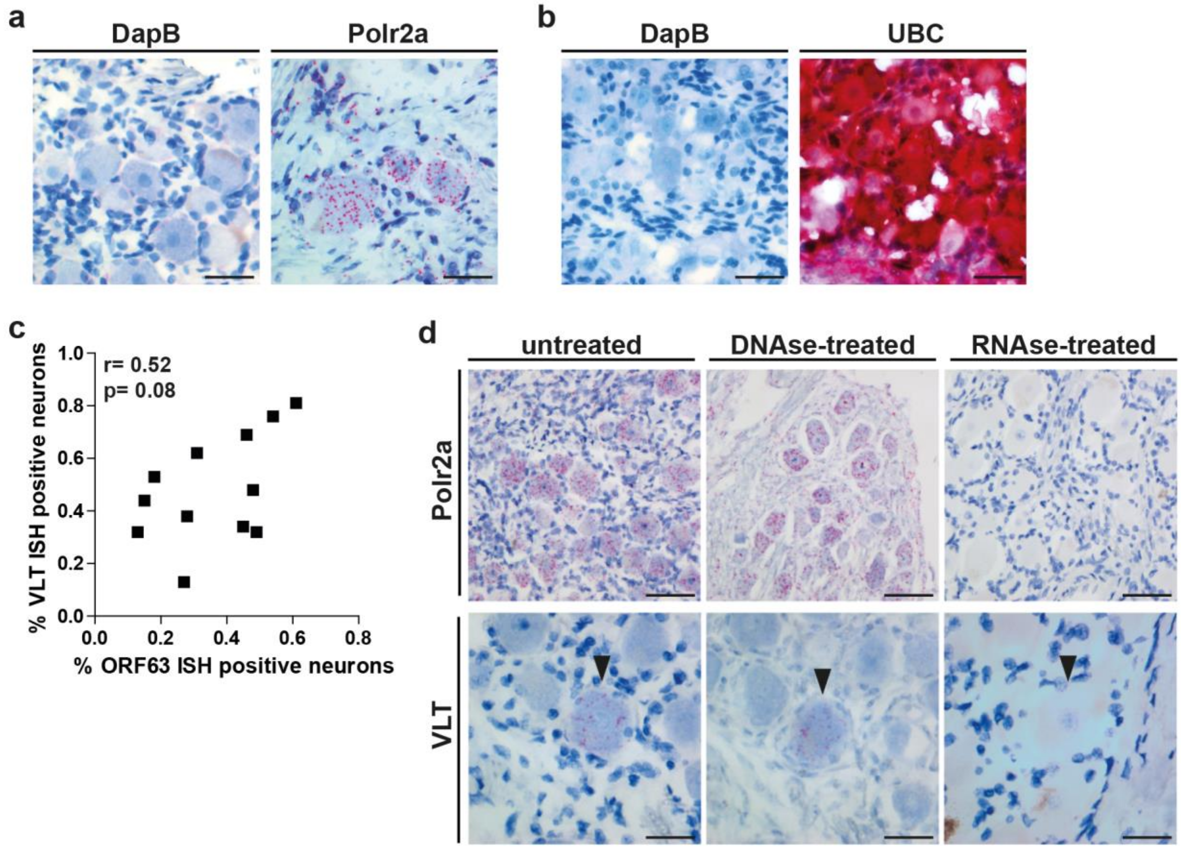


**Supplementary Figure 8 | VLT-specific probe detects viral RNA, but not viral DNA in human TG by *in situ* hybridization. a** and **b,** Analysis of negative control (bacterial gene dihydrodipicolinate reductase, DapB) and positive control [human gene RNA polymerase II polypeptide A (Polr2a) and ubiquitin C (UBC)] transcripts in human (**a**) latently VZV-infected adult TG and (**b**) VZV naïve fetal dorsal root ganglia by *in situ* hybridization (ISH). Magnification: 400X. Bars= 50µm. **c,** Correlation between the percentages of ORF63 RNA- and VLT-positive neurons in latently VZV-infected human TG, determined by ISH. Spearman correlation is indicated. **d,** Analysis of ISH probe specificity using untreated, DNase-treated or RNase-treated consecutive sections of human TG. Magnification: 200X (Polr2a) and 400X (VLT). Bars= 100µm (Polr2a) and 50µm (VLT). Arrowheads indicate the same neuron in adjacent TG sections. Representative images from two donors (n=2 sections/donor) are shown.

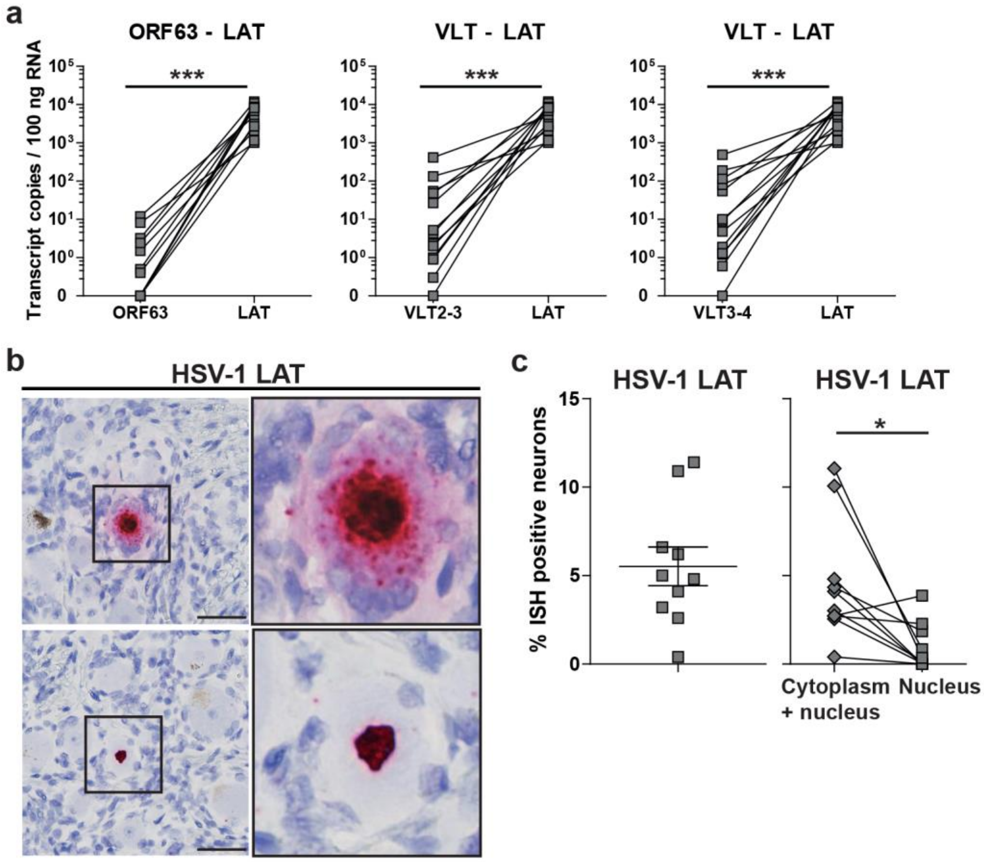


**Supplementary Figure 9 | Comparison of VZV transcripts and HSV-1 LAT in human TG. a,** RT-qPCR analysis of HSV-1 LAT, VZV ORF63 transcript and VLT levels in 13 latently VZV/HSV-1 co-infected human TG (Supplementary Table 1). VLT2-3 and VLT3-4 indicate primer/probe sets spanning VLT exons 2→3 and 3→4 junctions (Supplementary Table 5). *** $p < 0.001$; Wilcoxon signed rank test. **b** and **c,** Analysis of HSV-1 LAT in ten latently HSV-1-infected human TG by ISH. **b,** Representative images of ISH analysis on human TG sections using probes specific for HSV-1 LAT, showing neuronal LAT staining in both the nucleus and cytoplasm (top panels) or solely nuclear (bottom panels). Magnification: 400X; inset: 400X and 3X digital zoom. Bars= 50µm. **c,** Frequency of neurons expressing HSV-1 LAT (left panel), and frequency of neurons expressing LAT in both the nucleus and cytoplasm or only in the nucleus. Horizontal line and error bars indicate average ± SEM. * $p<0.05$; paired Student's t-test.

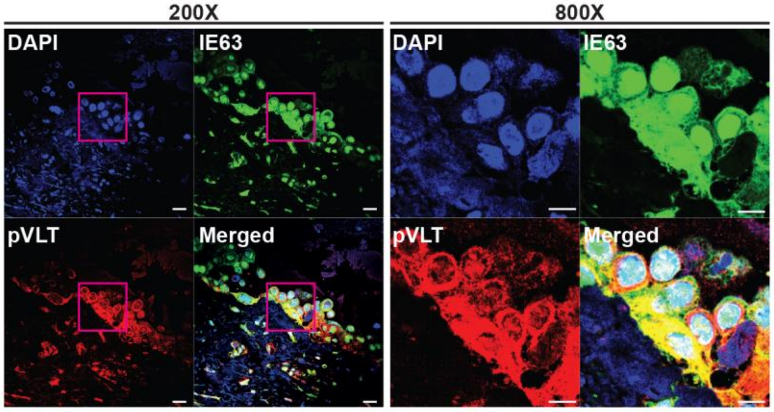


**Supplementary Figure 10 | Expression of VZV ORF63 and VLT protein in human herpes zoster skin biopsies.** Confocal microscopy image showing expression of protein encoded by VZV ORF63 (IE63; green) and VLT (pVLT; red) in the same cells of a human herpes zoster vesicle. Nuclei were counterstained with DAPI (blue). Magnification is indicated. Bars=20 μm (200X) and 10 μm (800X).

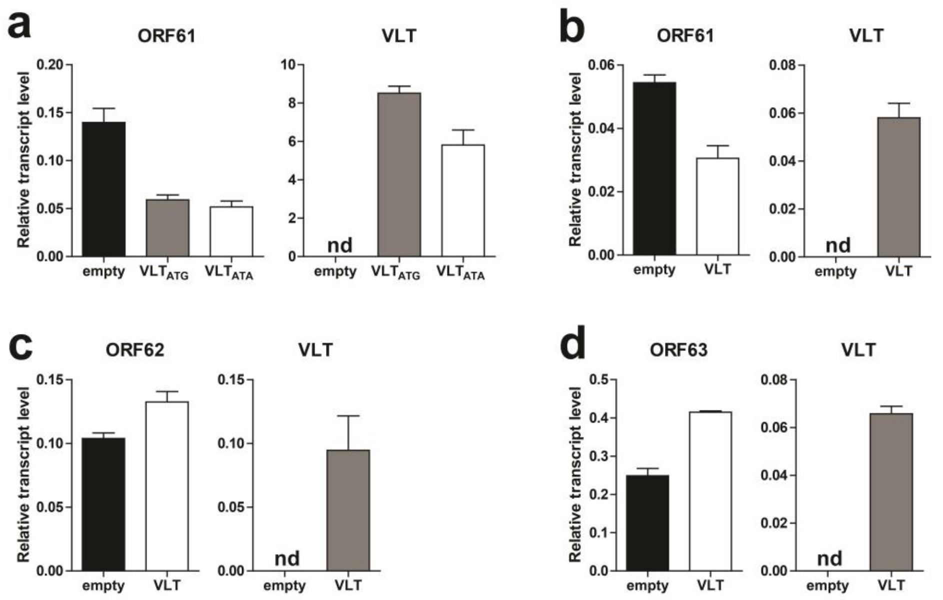


**Supplementary Figure 11 | Selective repression of VZV ORF61 gene expression by VLT in co-transfected cells. a,** Analysis of VZV ORF61 transcript and VLT abundance by RT-qPCR. ARPE-19 cells were transiently transfected with plasmids encoding either FLAG-tagged VLT ($VLT_{ATG}$), a mutant VLT in which the ATG start codon was replaced by ATA sequence ($VLT_{ATA}$) or an empty vector control plasmid (empty), in combination with a plasmid encoding ORF61 (double transfection). **b-d,** Analysis of VZV ORF61, ORF62, ORF63 transcript and VLT abundance in ARPE-19 cells co-transfected with FLAG-tagged VLT or empty vector control, and plasmids encoding ORF61 (**b**), ORF62 (**c**) or ORF63 (**d**). Relative transcript levels were determined as follows: $2^{-(\text{Ct-value VZV gene} - \text{Ct-value } \beta\text{-actin})}$. nd: not detected. Experiments were performed in duplicate and repeated twice. Representative duplicate measurements from one experiment are shown

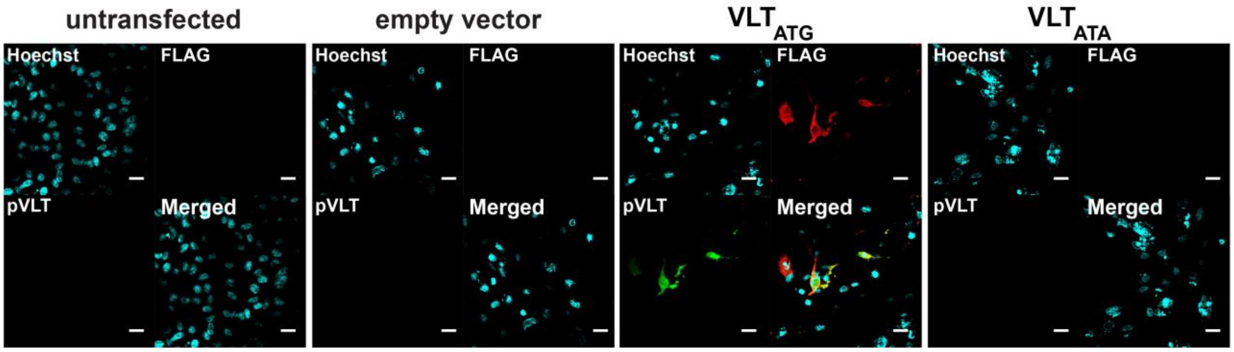


**Supplementary Figure 12 | Validation of VLT protein expression in co-transfection experiments.** Confocal microscopy image of untransfected ARPE-19 cells and of ARPE-19 cells transiently transfected with a plasmid encoding FLAG-tagged VLT ($VLT_{ATG}$), mutated VLT lacking a start codon ($VLT_{ATA}$) or empty control plasmid. Cells were stained using antibodies to FLAG (red) and VLT protein (pVLT; green); nuclei were stained with Hoechst 33342 (cyan). Magnification: 600X. Bars=20 μm.

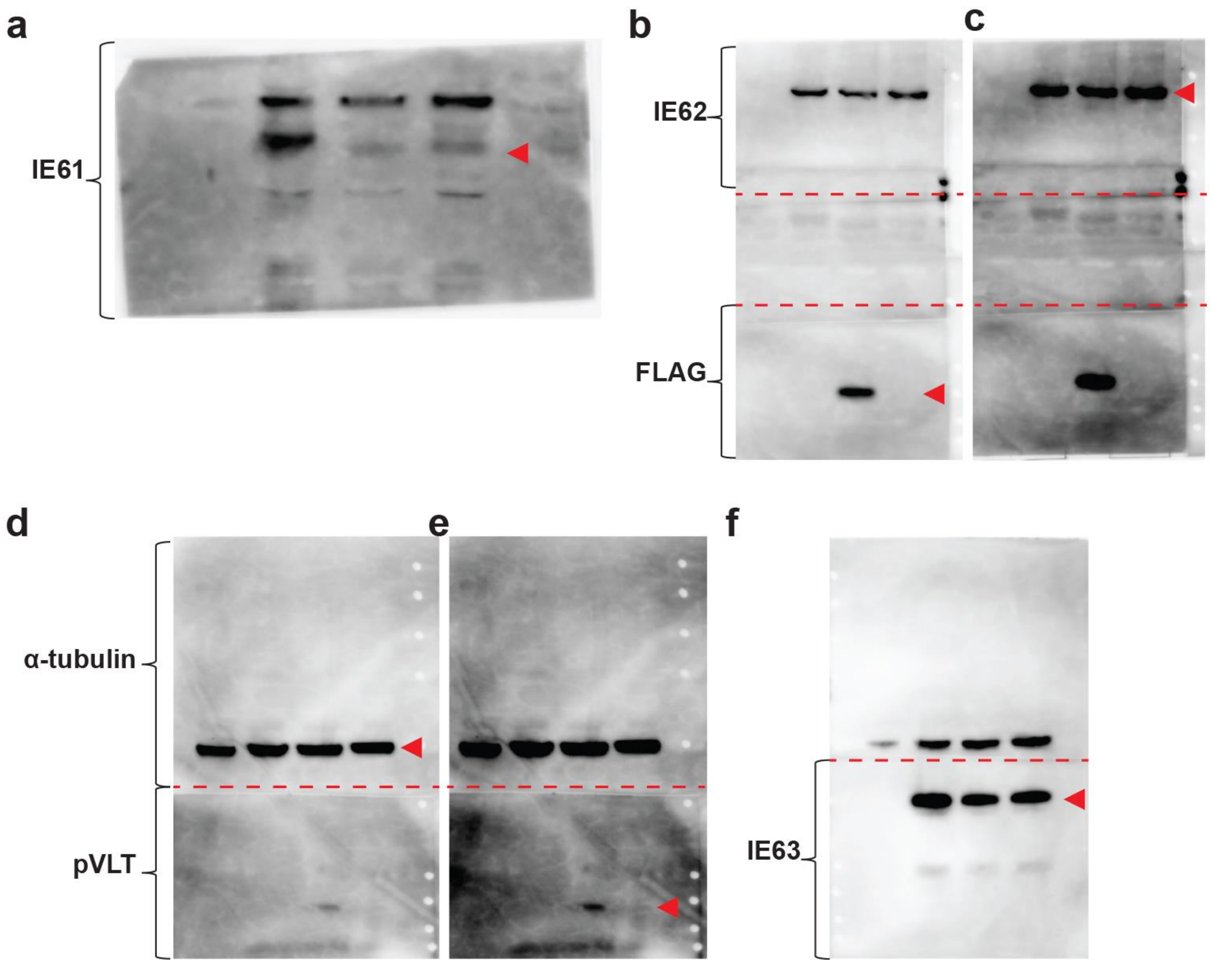


**Supplementary Figure 13 | Uncropped immunoblots for Figure 6b.** Membranes with transferred protein were cut (dashed red line above cutting line) before probing with appropriate primary antibodies and reassembled after reaction with chemiluminescence detection reagent. Curly brackets indicate region of membrane probed with a single antibody, red arrowheads bands at appropriate molecular weight as shown in Figure 6b. Primary antibodies used: (a) IE61, (b) and (c) IE62 and FLAG, (d) and (e) α-tubulin and pVLT and (f) IE63. Figures (b) + (c) and figures (d) + (e) represent the same membrane with adjusted brightness and contrast.

## Supplementary Tables

### Supplementary Table 1. Clinical and virological parameters of human trigeminal ganglion donors used for RNA-sequencing and qPCR.

| Donor[a] | Age (yrs) | Gender | Cause of death | Neurological Disease | PMI[b] (hr:min) | virus gec/$10^5$ TG cells[c] | | Virus transcript levels/100 ng RNA[d] | | | |
|---|---|---|---|---|---|---|---|---|---|---|---|
| | | | | | | VZV | HSV-1 | ORF63 | VLT2→3 | VLT3→4 | LAT |
| **1** | 93 | Fem | Respiratory insufficiency | Alzheimer's Disease | 5:20 | 30 | 30 | 0 | 1.0 | 1.9 | 8606 |
| **2** | 67 | Fem | Heart failure | Alzheimer's Disease | 6:45 | 249 | 26 | 3.2 | 26.8 | 54.7 | 11924 |
| **3** | 81 | Male | Decompensatio cordis | Parkinson's Disease | 5:15 | 164 | 23 | 1.5 | 54.4 | 79.7 | 2078 |
| **4** | 90 | Fem | Sudden cardiac arrest | Alzheimer's Disease | 4:45 | 91 | 7 | 11.9 | 412.4 | 489.0 | 5047 |
| **5** | 67 | Male | Euthanasia | Alzheimer's Disease | 8:00 | 28 | 29 | 0.5 | 4.3 | 9.0 | 8869 |
| **6** | 89 | Male | Sepsis | Alzheimer's Disease | 4:56 | 188 | 0[e] | 8.2 | 131.9 | 188.7 | 1004 |
| **7** | 73 | Fem | Heart failure | Parkinson's Disease | 4:40 | 99 | 13 | 2.4 | 48.4 | 114.7 | 6906 |
| **8** | 83 | Fem | Urosepsis and aspiration pneumonia | Other Neurological Disease | 5:20 | 43 | 553 | 0 | 0.3 | 0.6 | 10739 |
| **9** | 71 | Male | Cardiovascular accident | Parkinson's Disease | 5:05 | 84 | 5 | 0 | 2.3 | 4.8 | 1161 |
| **10** | 71 | Fem | Dehydration and cachexia | Alzheimer's Disease | 4:15 | 66 | 0 | 0.4 | 12.1 | 17.4 | 0 |
| **11** | 69 | Male | Dehydration and cachexia | Alzheimer's Disease | 6:05 | 84 | 119 | 0 | 5.2 | 10.0 | 3094 |
| **12** | 47 | Fem | Euthanasia | Non-demented control | 6:05 | 494 | 0 | 0.3 | 3.6 | 3.3 | 0 |
| **13** | 66 | Male | Uremia/cachexia | Alzheimer's Disease | 7:00 | 26 | 29 | 0 | 0 | 0 | 8205 |
| **14** | 89 | Fem | Euthanasia | Non-demented control | 4:45 | 0[e] | 0 | 0 | 0.9 | 1.2 | 8648 |
| **15** | 80 | Male | Dehydration and cachexia | Other Neurological Disease | 6:35 | 0[e] | 0 | 0.4 | 2.0 | 1.3 | 2699 |
| **16** | 69 | Fem | Euthanasia | Other Neurological Disease | 4:30 | 0 | 42 | 0 | 0 | 0 | 6905 |
| **17** | 95 | Fem | Alzheimer's disease and dehydration | Alzheimer's Disease | 5:30 | 0 | 1 | 0 | 0 | 0 | 24 |
| **18** | 83 | Male | Aspiration pneumonia | Multiple sclerosis | 7:50 | 0 | 10 | 0 | 0 | 0 | 1 |

[a] Donors 1 – 7 were used for RNA-sequencing; donors 1, 3 and 5 were used for miRNA-sequencing; donors 1 – 18 were used for quantitative real-time PCR (qPCR)
[b] PMI: post-mortem interval
[c] Viral DNA load in genome equivalent copies (gec) per $10^5$ trigeminal ganglia (TG) cells, as determined by virus-specific and single copy gene hydroxymethylbilane synthase (HMBS)-specific real-time PCR on DNA isolated from about one-fifth part of each TG specimen.
[d] Viral transcript copies per 100 ng total RNA isolated from about four-fifth part of each TG specimen. LAT, HSV-1 latency-associated transcript; ORF63, VZV open reading frame 63; VLT2→3 and VLT3→4, VZV latency-associated transcript (VLT) fragment detected by reversed transcribed real-time PCR using specific primer/probe-combinations.
[e] Individual TG were cut so that approximately one-fifth TG was used for DNA isolation and four-fifth was used for RNA isolation. Occasionally, discrepancies between viral DNA and cDNA qPCRs were observed, most likely due to unequal distribution of HSV-1 among TG neurons and low number of neurons harboring latent VZV. TG positive for either viral DNA or RNA were considered positive for the respective virus, while TG in which no viral nucleic acids could be detected were considered negative for the virus.

## Supplementary Table 2. RNA-sequencing metrics.

| RNA source[a] | Protocol[b] | Enrichment[c] | Read length (bp) | Total read pairs | Varicella-zoster virus (VZV) | | | | Herpes simplex virus type 1 (HSV-1) | | | |
|---|---|---|---|---|---|---|---|---|---|---|---|---|
| | | | | | Mapping read pairs | % | Diversity index[d] | De-duplicated read pairs | Mapping read pairs | % | Diversity index[d] | De-duplicated read pairs |
| TG #1 | polyA | VZV & HSV-1 | 2x75 | 49,173,713 | 7,180 | 0.01 | 0.975 | 181 | 20,937,718 | 42.58 | 0.998 | 46,831 |
| | polyA | None | 2x75 | 99,471,313 | 0 | 0.00 | 0.000 | 0 | 1,754 | 0.00 | 0.131 | 1,524 |
| | rRNA | VZV & HSV-1 | 2x75 | 50,935,304 | 5,939 | 0.01 | 0.997 | 18 | 27,548,446 | 54.09 | 0.997 | 81,254 |
| | rRNA | None | 2x75 | 50,215,904 | 0 | 0.00 | 0.000 | 0 | 843 | 0.00 | 0.069 | 785 |
| TG #2 | polyA | VZV & HSV-1 | 2x75 | 48,348,467 | 49,116 | 0.10 | 0.998 | 108 | 24,815,982 | 51.33 | 0.998 | 56,477 |
| | polyA | None | 2x75 | 90,146,278 | 0 | 0.00 | 0.000 | 0 | 1149 | 0.00 | 0.128 | 1,002 |
| | rRNA | VZV & HSV-1 | 2x75 | 46,504,366 | 31,910 | 0.07 | 0.998 | 53 | 27,744,406 | 59.66 | 0.998 | 55,857 |
| | rRNA | None | 2x75 | 65,995,396 | 0 | 0.00 | 0.000 | 0 | 1071 | 0.00 | 0.105 | 959 |
| TG #3 | polyA | VZV & HSV-1 | 2x150 | 92,777,528 | 408,427 | 0.44 | 0.998 | 829 | 7,837,812 | 8.45 | 0.997 | 23,058 |
| TG #4 | polyA | VZV & HSV-1 | 2x150 | 87,656,593 | 894,765 | 1.02 | 0.999 | 983 | 11,540,872 | 13.17 | 0.998 | 22,174 |
| TG #5 | polyA | VZV & HSV-1 | 2x150 | 72,643,442 | 53,030 | 0.07 | 0.992 | 414 | 16,463,232 | 22.66 | 0.992 | 81,607 |
| TG #6 | polyA | VZV & HSV-1 | 2x150 | 107,702,307 | 480,559 | 0.45 | 0.997 | 1,319 | 28,815,264 | 26.75 | 0.995 | 130,424 |
| TG #7 | polyA | VZV & HSV-1 | 2x150 | 91,143,646 | 212,995 | 0.23 | 0.998 | 351 | 10,423,148 | 11.44 | 0.996 | 36,490 |
| ARPE-19/ HSV-1 #1 | polyA | HSV-1 | 2x34 | 20,000,000[e] (79,677,197) | 275 | 0.00 | 0.404 | 164 | 19,543,817 | 97.72 | 0.789 | 4,117,726 |
| | polyA | None | 2x34 | 20,000,000[e] (32,199,707) | 86 | 0.00 | 0.291 | 61 | 13,192,165 | 65.96 | 0.806 | 2,564,304 |
| ARPE-19/ HSV-1 #2 | rRNA | HSV-1 | 2x34 | 20,000,000[e] (76,360,350) | 243 | 0.00 | 0.325 | 164 | 19,487,687 | 97.44 | 0.795 | 3,987,408 |
| | rRNA | None | 2x34 | 20,000,000[e] (26,101,860) | 205 | 0.00 | 0.063 | 192 | 15,905,450 | 79.53 | 0.836 | 2,601,054 |
| ARPE-19/ VZV #1 | polyA | VZV | 2x34 | 20,000,000[e] (62,922,757) | 18917428 | 94.59 | 0.810 | 3,598,314 | 419 | 0.00 | 0.468 | 223 |
| | polyA | None | 2x34 | 20,000,000[e] (44,024,427) | 3,759,823 | 18.80 | 0.691 | 1,162,827 | 57 | 0.00 | 0.070 | 53 |

[a] RNA was isolated from human trigeminal ganglia (TG) or human retina pigmented epithelial line ARPE-19 cells lytically infected with HSV-1 (ARPE-19/HSV-1) or VZV (ARPE-19/VZV). TG coding refers to donor ID presented in Supplementary table 1.
[b] Protocol refers to total RNA selected for polyadenylated RNA (polyA) or depleted for ribosomal RNA (rRNA).
[c] RNA libraries were enriched for HSV-1- and/or VZV-specific sequences or left untreated (none).
[d] Diversity index represents the complexity of the library (in other words, the level of read duplicates that can be attributed to PCR is calculated using PicardTools RemoveDuplicates).
[e] Sequence datasets were subsampled to 20,000,000 read-pairs due to facilitate mapping of VZV and HSV-1 reads. Total numbers of read-pairs are showing in brackets.

**Supplementary table 3. Location and nucleotide sequence of the exons and introns of varicella-zoster virus latency transcript.**

| | Start[a] | End | Length[b] | Sequences[c] |
|---|---|---|---|---|
| ***Exon 1*** | 102,421 | 102,483 | 63 | ACTATCCAGT TGGCATTTTA AACGGGTCCG GCTGCCTAAA CCGAAAACAC CGTTGCCTTT ACT |
| ***Intron 1*** | 102,484 | 102,853 | 370 | ***GT***AAGTACAA AACTAAAATT TATATTTGCG …… GTTAGACTGT TATGTTTATT GTATTTGC***AG*** |
| ***Exon 2*** | 102,854 | 102,982 | 129 | AGCAGG***ATG***C CCCGGTTACT CCGAGACCGG …… CCAACCCTTA CGACCAATAG CAACACTCAG |
| ***Intron 2*** | 102,983 | 103,827 | 845 | ***GT***ATTTTTAA AATGCACGTT TAATGATCAT …… ATGGTTCGTG GGTCTTGATT AATTCCCAC***AG*** |
| ***Exon 3*** | 103,828 | 103,923 | 96 | ATACTGGACG ATCACGGTAG TCCTGCCCCC …… ACACCACCGG GGTCGCCGAT CGAACAGCAG |
| ***Intron 3*** | 103,924 | 104,293 | 370 | ***GT***TGGTCTTT AAAAAATACC TTCCGTAAAA …… GTAACTTGTA TCACTTACGA TCTTATGC***AG*** |
| ***Exon 4*** | 104,294 | 104,432 | 139 | GATGGATTGC ACTGGACACC GGCAGAGAGG …… TCGGAAACGG TGCTCATGCA TATGGTGCAG |
| ***Intron 4*** | 104,433 | 104,768 | 336 | ***GT***ATTATCCG AAGCGTCGGA GGTGCCGCTA …… AGTGACATTT TAGATTCTGT CTTTATTT***AG*** |
| ***Exon 5*** | 104,769 | 104,837 | 69 | ATAAAGAGCG ATACGAAGAC ATTTCTCCAC CCCCCTGTAA TACCCGTAAA ***TAA***AGGTAAG TCCACAAAC |

[a] Coordinates of VLT exons and introns with respect to VZV reference strain Dumas (NCBI Reference Sequence: NC_001348.1).
[b] Sequence length in base pairs.
[c] ***GT***: splice donor site; ***AG***: splice acceptor site; ***ATG***: start codon; ***TAA***: stop codon.

**Supplementary Table 4. Single Nucleotide Polymorphisms (SNPs) across the VLT locus[a]**

| Genome position[b] | Reference base[b] | Variant base | ORF | Type of mutation | Codon position | Mutation | Number of genomes[c] | Note |
|---|---|---|---|---|---|---|---|---|
| 102002 | T | C | n.a. | non-coding | . | . | 1 (0) | |
| 102232 | C | A | n.a. | non-coding | . | . | 1 (0) | |
| 102268 | T | C | n.a. | non-coding | . | . | 1 (0) | |
| 102309 | C | A | n.a. | non-coding | . | . | 21 (6) | |
| 102351 | A | C | n.a. | non-coding | . | . | 19 (6) | |
| 102401 | G | A | n.a. | non-coding | . | . | 1 (0) | VLT exon 1 |
| 102403 | A | G | n.a. | non-coding | . | . | 1 (0) | VLT exon 1 |
| 102403 | A | C | n.a. | non-coding | . | . | 8 (0) | VLT exon 1 |
| 102458 | A | G | n.a. | non-coding | . | . | 21 (6) | VLT exon 1 |
| 102510 | T | C | n.a. | non-coding | . | . | 2 (0) | VLT intron |
| 102575 | A | G | n.a. | non-coding | . | . | 2 (0) | VLT intron |
| 102596 | C | A | n.a. | non-coding | . | . | 1 (0) | VLT intron |
| 102601 | T | G | n.a. | non-coding | . | . | 19 (6) | VLT intron |
| 102617 | C | T | n.a. | non-coding | . | . | 3 (0) | VLT intron |
| 102684 | A | T | n.a. | non-coding | . | . | 1 (0) | VLT intron |
| 102738 | T | C | n.a. | non-coding | . | . | 2 (0) | VLT intron |
| 102850 | G | A | n.a. | non-coding | . | . | 1 (1) | VLT intron |
| **102908** | **C** | **T** | **VLT** | **non-synonymous** | **1** | **R17T** | **1 (0)** | **VLT exon 2** |
| **102970** | **A** | **G** | **VLT** | **synonymous** | **3** | **Q37** | **2 (0)** | **VLT exon 2** |
| **102977** | **C** | **T** | **VLT** | **non-synonymous** | **1** | **H40T** | **1 (1)** | **VLT exon 2** |
| 103043 | T | C | n.a. | non-coding | . | . | 20 (6) | VLT intron |
| *103169* | *A* | *G* | *ORF61* | *synonymous* | *3* | *S439* | *1 (2)* | *VLT intron* |
| *103205* | *A* | *G* | *ORF61* | *synonymous* | *3* | *P427* | *1 (0)* | *VLT intron* |
| *103261* | *A* | *G* | *ORF61* | *non-synonymous* | *1* | *T409A* | *1 (0)* | *VLT intron* |
| *103295* | *C* | *T* | *ORF61* | *synonymous* | *3* | *G397* | *8 (0)* | *VLT intron* |
| *103322* | *T* | *C* | *ORF61* | *synonymous* | *3* | *G388* | *1 (0)* | *VLT intron* |
| *103613* | *G* | *T* | *ORF61* | *synonymous* | *3* | *A291* | *2 (0)* | *VLT intron* |
| *103754* | *A* | *G* | *ORF61* | *synonymous* | *3* | *G244* | *1 (0)* | *VLT intron* |
| *103778* | *A* | *G* | *ORF61* | *synonymous* | *3* | *R246* | *2 (0)* | *VLT intron* |
| *103799* | *C* | *T* | *ORF61* | *synonymous* | *3* | *T229* | *1 (0)* | *VLT intron* |
| *103805* | *A* | *G* | *ORF61* | *synonymous* | *3* | *P227* | *1 (0)* | *VLT intron* |
| *103955* | *A* | *C* | *ORF61* | *synonymous* | *3* | *S177* | *1 (0)* | *VLT intron* |
| *104047* | *G* | *A* | *ORF61* | *non-synonymous* | *1* | *A147T* | *1 (0)* | *VLT intron* |
| *104120* | *A* | *G* | *ORF61* | *synonymous* | *3* | *T122* | *1 (0)* | *VLT intron* |
| *104135* | *C* | *T* | *ORF61* | *synonymous* | *3* | *S117* | *1 (0)* | *VLT intron* |
| *104156* | *C* | *T* | *ORF61* | *synonymous* | *3* | *S110* | *1 (0)* | *VLT intron* |
| *104275* | *G* | *A* | *ORF61* | *non-synonymous* | *1* | *D71N* | *1 (0)* | *VLT intron* |
| **104403** | **A** | **G** | **VLT** | **non-synonymous** | **2** | **D110G** | **1 (0)** | **VLT exon 4** |
| | *T* | *C* | *ORF61* | *synonymous* | *3* | *D28* | *1 (0)* | |
| **104406** | **G** | **A** | **VLT** | **non-synonymous** | **2** | **R111E** | **7 (0)** | **VLT exon 4** |
| | *C* | *T* | *ORF61* | *synonymous* | *3* | *S27* | *7 (0)* | |
| *104472* | *T* | *C* | *ORF61* | *non-synonymous* | *2* | *L5S* | *1 (0)* | *VLT intron* |
| *104477* | *C* | *T* | *ORF61* | *synonymous* | *3* | *T3* | *1 (0)* | *VLT intron* |
| 104684 | A | C | n.a. | non-coding | . | . | 1 (0) | |
| 104723 | G | T | n.a. | non-coding | . | . | 1 (1) | |
| 104737 | T | G | n.a. | non-coding | . | . | 1 (0) | |
| 104738 | A | C | n.a. | non-coding | . | . | 2 (0) | |
| 104749 | T | C | n.a. | non-coding | . | . | 1 (0) | |
| 104865 | G | A | n.a. | non-coding | . | . | 1 (0) | |
| 104877 | T | G | n.a. | non-coding | . | . | 1 (0) | |
| 104882 | C | A | n.a. | non-coding | . | . | 11 (0) | |
| 104898 | A | G | n.a. | non-coding | . | . | 84 (6) | |
| 104913 | G | A | n.a. | non-coding | . | . | 1 (0) | |
| 104933 | G | A | n.a. | non-coding | . | . | 4 (0) | |
| 104939 | C | - | n.a. | non-coding | . | . | 1 (0) | |

[a] SNPs within the coding region of VLT are shown in bold and within ORF61 are shown using italics.
[b] Reference bases and genome co-ordinates correspond to VZV reference strain Dumas NC_001348.1
[c] Data originate from a multiple sequence alignment of VZV wildtype (n = 88) and vaccine / vaccine rash (n = 38) strains obtained from Genbank in August 2017. The numbers of wildtype and (vaccine rash) genomes carrying the SNP are reported.

**Supplementary Table 5. Primers and probes.**

| Name[a] | Target[b] | Sequence (5' → 3') | Modifications[c] | |
|---|---|---|---|---|
| | | | 5' | 3' |
| VLT2-3_Probe | VZV VLT splice junction exons 2→3 | GCA-ACA-CTC-AGA-TAC-TGG-ACG-ATC | 6-FAM | BHQ1 |
| VLT2-3_Fw | VZV VLT exon 2 | GAC-TTA-CCA-GGG-GGC-AGT-AT | none | none |
| VLT2-3_Rv | VZV VLT exon 3 | CGG-TGG-TGT-ATG-GCT-CGA-TT | none | none |
| VLT3-4_Probe | VZV VLT splice junction exons 3→4 | GAT-CGA-ACA-GCA-GAT-GGA-TTG-CAC | 6-FAM | BHQ1 |
| VLT3-4_Fw | VZV VLT exon 3 | AAT-CGA-GCC-ATA-CAC-CAC-CG | none | none |
| VLT3-4_Rv | VZV VLT exon 4 | TGT-ATT-CGG-GCA-TGG-ACC-TC | none | none |
| ORF62_Probe | VZV ORF62 | TGC-AAC-CCG-GGC-GTC-CG | 6-FAM | BHQ1 |
| ORF62_Fw | VZV ORF62 | CCT-TGG-AAA-CCA-CAT-GAT-CGT | none | none |
| ORF62_Rv | VZV ORF62 | AGC-AGA-AGC-CTC-CTC-GAC-AA | none | none |
| ORF63_Probe | VZV ORF63 | TGT-CCC-ATC-GAC-CCC-CTC-GG | 6-FAM | BHQ1 |
| ORF63_Fw | VZV ORF63 | GCT-TAC-GCG-CTA-CTT-TAA-TGG-AA | none | none |
| ORF63_Rv | VZV ORF63 | GCC-TCA-ATG-AAC-CCG-TCT-TC | none | none |
| HMBS_probe | Human hydroxymethylbilane synthase | TGG-AAG-CTA-ATG-GGA-AGC-CCA-GTA-CC | 6-FAM | TAMRA |
| HMBS_Fw | Human hydroxymethylbilane synthase | GCC-TGC-AGT-TTG-AAA-TCA-GTG | none | none |
| HMBS_Rv | Human hydroxymethylbilane synthase | CGG-GAC-GGG-CTT-TAG-CTA | none | none |
| ORF29F2381 | VZV ORF29 | GCC-TTG-CAA-GTG-CGT-ACC | none | none |
| ORF29R2440 | VZV ORF29 | CTA-GGG-CCC-CGT-GTA-ACA-TA | none | none |
| VLTexon1F | VZV VLT exon 1 | GGC-ATT-TTA-AAC-GGG-TCC-GG | none | none |
| VLTexon2R-1 | VZV VLT exon 2 | CCC-TGG-TAA-GTC-CGT-ACA-CG | none | none |
| VLTup22ecoF | VZV VLT | ACC-GAA-TTC-CAC-CGT-TGC-CTT-TAC-TAG-CAG | none | none |
| VLT-FLAGxhoR | VZV VLT | ACC-CTC-GAG-TTA-CTT-GTC-GTC-ATC-GTC-TTT-GTA-GTC-TTT-ACG-GGT-ATT-ACA-GGG | none | none |
| VLT-G3A | VZV VLT | TAT-TTG-CAG-AGC-AGG-ATA-CCC-CGG-TTA-CTC-CGA | none | none |
| ORF61up20ecoF | VZV ORF61 | ACC-GAA-TTC-GAA-TAC-AGC-CAG-TTG-TTA-CC | none | none |
| ORF61salR | VZV ORF61 | ACC-GTC-GAC-CTA-GGA-CTT-CTT-CAT-CTT-GTT-TGG | none | none |
| ORF63up20exoF | VZV ORF63 | ACC-GAA-TTC-GTT-ACT-ACG-GCC-CCA-AGG | none | none |
| ORF63xhoR | VZV ORF63 | ACC-CTC-GAG-CTA-CAC-GC-CAT-GG | none | none |
| VLTexon2F | VZV VLT exon 2 | CAG-GAT-GCC-CCG-GTT-ACT-C | none | none |
| VLTexon2R-2 | VZV VLT exon 2 | GCT-ATT-GGT-CGT-AAG-GGT-TGG | none | none |
| HHV1_LAT_Fw | HSV-1 LAT 2.0kb intron | CCC-ACG-TAC-TCC-AAG-AAG-GC | none | none |
| HHV1_LAT_Rv | HSV-1 LAT 2.0kb intron | AGA-CCC-AAG-CAT-AGA-GAG-CCA-G | none | none |
| HHV1_LAT_Probe | HSV-1 LAT 2.0kb intron | CCC-ACC-CCG-CCT-GTG-TTT-TTG-TG | 6-FAM | BHQ1 |
| NheI_VLT_F | VZV VLT | GCA-GCT-AGC-ATG-CCC-CGG-TTA-CTC-CG | none | none |
| VLT_R_BamHI | VZV VLT | CGT-GGA-TCC-TTA-TTT-ACG-GGT-ATT-ACA-GGG-G | none | none |
| ORF62F2016 | VZV ORF62 | TCC-ACC-GGA-TGA-TCG-TTT-AC | none | none |
| ORF62R2083 | VZV ORF62 | GGA-GGC-TTC-TGC-TCT-CGA-C | none | none |
| VLT_E-1A_Probe | VZV VLT splice junction exon A → exon1 | CAA-AGC-ATC-GCA-CTA-TCC-AGT-TGG | 6-FAM | BHQ1 |
| VLT_E-1A_Fw | VZV VLT exon A | CCA-TAC-ACC-GGA-AAG-GGC-AT | none | none |
| VLT_E-1B_Probe | VZV VLT splice junction exon B → exon1 | GGA-AGC-ATG-GAC-AAC-TAT-CCA-GTT-G | 6-FAM | BHQ1 |

| | | | | |
|---|---|---|---|---|
| VLT_E-1B_Fw | VZV VLT exon B | TGT-TTT-CCC-TTC-ACT-ACG-ACG-T | none | none |
| VLT_E-1C_Probe | VZV VLT splice junction exon C → exon1 | AGA-TTG-ATT-TGC-GAC-TAT-CCA-GTT-GG | 6-FAM | BHQ1 |
| VLT_E-1C_Fw | VZV VLT exon C | CCC-AAC-GGC-CAT-TAT-CCC-TT | none | none |
| VLT_E1_Rv | VZV VLT exon 1 | AAA-GGC-AAC-GGT-GTT-TTC-GG | none | none |
| VLT2 | VZV VLT exon 2 | GCT-ATT-GGT-CGT-AAG-GGT-TGG | none | none |
| RACEout_Fw | 5'RACE Adapter | GCT-GAT-GGC-GAT-GAA-TGA-ACA-CTG | none | none |
| RACEin_Fw | 5'RACE Adapter | CGC-GGA-TCC-GAA-CAC-TGC-GTT-TGC-TGG-CTT-TGA-TG | none | none |
| Universal Primer A Mix | RACE Adapter (Mixture of Long and Short) | Long; CTA-ATA-CGA-CTC-ACT-ATA-GGG-CAA-GCA-GTG-GTA-TCA-ACG-CAG-AGT<br>Short; CTA-TAG-GGC-AAG-CAG-TGG-TAT-CAA-CGC-AGA-GT | none | none |
| ORF61R626AS | Anti-sense to VZV ORF61 (VLT exon 3) | CCG-GGG-GGC-AGG-ACT-ACC-GTG-AT | none | none |
| ORF61F131AS | Anti-sense to VZV ORF61 (VLT exon 4) | GAC-GCT-GGT-GGA-GGT-CCA-TGC-CCG-AA | none | none |
| pOka104778R | VZV VLT exon 5 | ACC-CTC-GAG-TAC-GGG-TAT-TAC-AGG-G | none | none |
| M13_Fw | pCR4-TOPO TA vector | TGT-AAA-ACG-ACG-GCC-AGT | none | none |
| M13_Rv | pCR4-TOPO TA vector | CAG-GAA-ACA-GCT-ATG-ACC | none | none |
| ORF61_13 | VLT exon 1 | ACC-GAA-AAC-ACC-GTT-GCC-TTT | none | none |
| VLT_exon5_Rv | VLT exon 5 | GGG-GTG-GAG-AAA-TGT-CTT-CGT | none | none |

[a] VLT, varicella-zoster virus latency-associated transcript; ORF, open reading frame; Rv, reverse; Fw, forward.
[b] 5'RACE Adapter sequences from FirstChoice[TM] RLM-RACE Kit (Ambion).
[c] 6-FAM, 6-carboxyfluorescein; BHQ1, black hole quencher 1; TAMRA, tetramethylrhodamine.
