# Supplementary material for: A spliced latency-associated VZV transcript maps antisense to the viral transactivator gene 61
Source: Nat Commun. 2018 Mar 21;9:1167. doi: 10.1038/s41467-018-03569-2 (PMC5862956; doi:10.1038/s41467-018-03569-2)
Supplement: Supplementary file 3 — Description of Additional Supplementary Files(PDF 173 kb) [file 41467_2018_3569_MOESM3_ESM.pdf]

Description of Additional Supplementary Files

File Name: Supplementary Data 1
Description: The VZV RNA 120-mer oligonucleotide 'bait' designs used in this study. Bait sequences are shown in their native .tdt format that can be readily uploaded to Agilent SureDesign for synthesis. Columns are labelled as TargetID, ProbeID (must be unique), Sequence (the 120-mer sequence), and Replication (relative number of copies of the bait sequence).

File Name: Supplementary Data 2
Description: The HSV-1 RNA 120-mer oligonucleotide 'bait' designs used in this study. Bait sequences are shown in their native .tdt format that can be readily uploaded to Agilent SureDesign for synthesis. Columns are labelled as TargetID, ProbeID (must be unique), Sequence (the 120-mer sequence), and Replication (relative number of copies of the bait sequence).
